# Quantum Nonlocality and Quantum Correlations in the Stern–Gerlach Experiment

**DOI:** 10.3390/e20040299

**Published:** 2018-04-19

**Authors:** Alma Elena Piceno Martínez, Ernesto Benítez Rodríguez, Julio Abraham Mendoza Fierro, Marcela Maribel Méndez Otero, Luis Manuel Arévalo Aguilar

**Affiliations:** Facultad de Ciencias Físico Matemáticas, Benemérita Universidad Autónoma de Puebla, 18 Sur y Avenida San Claudio, Col. San Manuel, Puebla 72520, Mexico

**Keywords:** quantum nonlocality, quantum mechanics, Stern–Gerlach experiment

## Abstract

The Stern–Gerlach experiment (SGE) is one of the foundational experiments in quantum physics. It has been used in both the teaching and the development of quantum mechanics. However, for various reasons, some of its quantum features and implications are not fully addressed or comprehended in the current literature. Hence, the main aim of this paper is to demonstrate that the SGE possesses a quantum nonlocal character that has not previously been visualized or presented before. Accordingly, to show the nonlocality into the SGE, we calculate the quantum correlations C(z,θ) by redefining the Banaszek–Wódkiewicz correlation in terms of the Wigner operator, that is $C\left(z,\theta \right)=\langle \Psi |\hat{W}\left(z,p_{z}\right)\hat{\sigma }\left(\theta \right)|\Psi \rangle$, where $\hat{W}\left(z,p_{z}\right)$ is the Wigner operator, $\hat{\sigma }\left(\theta \right)$ is the Pauli spin operator in an arbitrary direction θ and |Ψ〉 is the quantum state given by an entangled state of the external degree of freedom and the eigenstates of the spin. We show that this correlation function for the SGE violates the Clauser–Horne–Shimony–Holt Bell inequality. Thus, this feature of the SGE might be interesting for both the teaching of quantum mechanics and to investigate the phenomenon of quantum nonlocality.

## 1. Introduction

The Stern–Gerlach experiment (SGE) [1,2,3,4] has played an important role in both the teaching and advancement of quantum mechanics. In quantum physics, this experiment is commonly used to introduce the concept of the internal spin of quantum systems, which has no counterpart in classical systems. Although nowadays, a significant number of new proposals has arisen to enhance our general understanding of the way it works and its possible uses [5,6,7,8,9,10,11,12,13,14], nonetheless, after almost one hundred years since its inception, still, there is not a total understanding of how it works yet in terms of an entire quantum mechanical description [4,5,6,7,8,9,10,11,12,13,14,15,16]; also, see [17]. In fact, most research analyses and textbooks have regularly focused on an SGE’s semiclassical model [5,6,9]. As an example of new approaches, Boustimi et al. gave a step forward by abandoning the usual magnets’ configuration producing a quadrupolar static magnetic field (by using just four bars) with the objective of modulating an atomic beam by means of an interference pattern [18]; see also [19]. Additionally, Machluf et al. have produced a field gradient beam splitter to create a coherent momentum superposition for matter-wave interferometry [20]. Of high relevance, the SGE was proposed as a system for implementing quantum roulettes, which are generalized quantum measurements [12]. In this case, a fluctuating magnetic field induces a probability distribution, which is used to implement a positive operator-value measure (POVM), which describes a continuous quantum roulette; for details, see [12].

Recently, we argued that the SGE could easily be used to introduce the concept of entanglement between the external and internal degrees of freedom in the teaching of quantum mechanics [5,6], to exemplify the entanglement generation between discrete and continuous variables and between pure and mixed states, as well. To show these properties of the SGE, we have used the evolution operator method [21,22,23,24,25]; to see an independent test of this method, see [26,27].

As we stated above, one of the main goals of the present paper is to show that the SGE possesses nonlocal correlations between internal and external degrees of freedom. In this way, this finding, of the SGE’s nonlocality features, will serve to stress its paramount importance in teaching quantum mechanics, and likewise, it might open a new avenue for investigation and the understanding of this famous experiment. In fact, the physical education research community (PER) is currently undergoing intense research and development regarding the learning and teaching of quantum mechanics, where it is important to highlight the importance of SGE [28,29,30,31,32,33,34,35,36,37,38,39,40,41,42,43,44,45,46,47,48,49,50,51,52,53].

## 2. Quantum Nonlocality

As was indicated by Clauser and Shimony [54], realism, which is the philosophical conception held by most physicists, claims that the external reality is supposed to have definite properties, i.e., predetermined values, independently of whether or not they are “observed”; this seems to support the objectivity of scientific investigation about nature. However, some of the quantum mechanics implications represent a direct challenge to this conception, e.g., the superposition principle. Particularly, the nonlocal character of quantum mechanics seems to imply that reality is not as direct as it was previously thought; instead, it is “created” by the measurement process. For example, if we get the singlet spin state ${|\psi \rangle }_{AB}=\frac{1}{\sqrt{2}}\left({|{↑}_{z}\rangle }_{A}{|{↓}_{z}\rangle }_{B}-{|{↓}_{z}\rangle }_{A}{|{↑}_{z}\rangle }_{B}\right)$, where ${|{↑}_{z}\rangle }_{A}$ (${|{↓}_{z}\rangle }_{A}$) represents spin up (spin down) of system *A* in the *z* direction, and we separate each of its two parts very far apart, when measuring the observable ${\hat{\sigma }}_{z}$ on part *A*, then part *B* acquires a defined *z* spin component. If, instead of measuring the observable ${\hat{\sigma }}_{z}$, we decide to measure the observable ${\hat{\sigma }}_{y}$ on *A*, then part *B* will acquire a definite *y* spin component. Therefore, by choosing to measure observable ${\hat{\sigma }}_{z}$ or ${\hat{\sigma }}_{y}$ on system *A*, a property of system *B* is “created”; this is only possible because the nonlocal character of quantum mechanics allows nonlocal correlations [55], since the systems are very far apart.

Hence, quantum nonlocality is a valuable resource, which allows the performance of many non-classical tasks, and at first instance, it was believed to be equivalent to entanglement [56]. However, nowadays, it is understood that nonlocality and entanglement agree with each other when the entangled systems are in pure states only, because it was proven that Bell’s inequality holds for all non-product states [57]; that is to say, any entangled state possesses quantum correlations that result in a contradiction with local classical theories [58]. Additionally, there are mixed entangled states whose quantum correlations could be explained by means of local hidden variables theories, and they could still be used to implement probabilistic teleportation protocols [59], i.e., quantum entanglement differs from quantum nonlocality [60,61].

Furthermore, one way of perceiving differences between entanglement and nonlocality is by demonstrating that there exist sets of quantum unentangled states that, however, possess nonlocal correlations in the sense that they may not be reliably distinguished by local measurements on the parts, and neither may the cloning operation be implemented by local operations on them [61].

Historically, the Fifth Physical Conference of the Solvay Institute, held in 1927, was one of the first times when the counterintuitive features of quantum nonlocality were addressed. In this conference, Einstein put forward a thought experiment, now called *Einstein’s boxes*, where he uncovers an important facet of the nonlocal character of quantum mechanics [62]. In this experiment, a single particle wave function is diffracted by a single slit, and the ongoing spherical wave function given by ψ(x,t) is spread over a hemisphere screen; then, according to Einstein, ${\left|\psi \left(x,t\right)\right|}^{2}$ expresses the probability that, at a given moment, the particle arrives at an arbitrary point belonging to the hemisphere screen [62]. Therefore, to rule out the possibility of being located at more than one place, there must be an instantaneous action on the entire screen. Jammer’s translation of Einstein’s words says: “a peculiar action-at-a-distance must be assumed to take place which prevent the continuously distributed wave in space from producing an effect at two places on the screen” [62]. This thought experiment was further stated in terms of two boxes by Einstein in a letter addressed to Schrödinger [63,64]; this letter seems to be the source of Schrödinger’s cat paradox [63]. Probably the second occasion where the counterintuitive nonlocal feature of quantum mechanics emerged was in the Einstein et al. paper of 1935, where the Einstein-Podolsky-Rosen paradox was established [65]. Nowadays, quantum nonlocality is believed to be different from entanglement, and it is taken as another quantum resource. Like entanglement, nonlocality cannot be created by local operations and classical communications [66]. Furthermore, the nonlocality resource can be distilled, in a similar way as entanglement [67]. Consequently, it is important to extensively study nonlocality for the sake of a better understanding of its relation with entanglement.

## 3. The SGE in A Complete Quantum Treatment

The SGE experiment is usually analyzed in most textbooks in a semiclassical way, where the external degrees of freedom are considered as classical variables, and its dynamics is thought of in terms of Newton’s second law [5,6,9]. However, in the scientific literature, we can find proposals that treat the external degrees of freedom (EDF) as a quantum variable giving a quantum description of the evolution of the EDF; see [5,6,7,8,9,10,11,13]. Nevertheless, in a recent paper, we gave a complete quantum treatment of the SGE [5,6], as it was originally thought of by Scully et al. [9], by using the evolution operator method [21,22,23,24,25] and obtaining the solution to the Schrödinger equation; this allows us to see the quantum features of the EDF. It is worth mentioning that in Figure 1 of [5], we are replacing the usual continuous path by a dotted one to stress the absence of classical paths. Then, one of our aims in this paper is to extract the nonlocal implications of the quantum treatment of the SGE.

For the case of the evolution of an initial superposition state of the spin degree of freedom, we have the initial state:

(1) $$|\psi (0)\rangle =\psi_{0}\left(\alpha |{↑}_{z}\rangle +\beta |{↓}_{z}\rangle \right),$$

where α and β are constants obeying ${\left|\alpha \right|}^{2}+{\left|\beta \right|}^{2}=1$, and $\psi_{0}$ is the Gaussian wave packet:

(2) $$\psi_{0}=\frac{1}{{\left(2\pi \sigma_{0}^{2}\right)}^{3/4}}\exp\left(-\frac{r^{2}}{4\sigma_{0}^{2}}+ik\cdot r\right),$$

where $\sigma_{0}$ is the width of the wave packet, r is the position and k is the wave vector. Then, the evolved state is given by:

(3) $$\begin{array}{ccc} |\psi (t)\rangle & = & e^{-\frac{it}{\hbar }\left(\frac{-\hbar^{2}}{2m}\nabla^{2}+\mu_{c}\left(\hat{\sigma }\cdot B\right)\right)}[\psi_{0}{(\alpha |↑}_{z}\rangle +\beta |{↓}_{z}\rangle )]\\ & = & \exp\left(\frac{-it^{3}\mu_{c}^{2}b^{2}}{6m\hbar }\right){\left[\frac{\sigma_{0}}{{\left(2\pi \right)}^{1/2}}\right]}^{3/2}{\left(\sigma_{0}^{2}+\frac{i\hbar t}{2m}\right)}^{-3/2}\exp\left(-\sigma_{0}^{2}k_{y}^{2}\right)\\ & \times & \exp\left[\frac{i4y\sigma_{0}^{2}k_{y}}{4(\sigma_{0}^{2}+it\hbar /2m)}\right]\exp\left[\frac{-(x^{2}+y^{2}-4\sigma_{0}^{4}k_{y}^{2})}{4(\sigma_{0}^{2}+it\hbar /2m)}\right]\\ & \times & \left\{\alpha \exp\left[\frac{-it\mu_{c}}{\hbar }\left(B_{0}+bz\right)\right]\exp\left[\frac{-1}{4(\sigma_{0}^{2}+it\hbar /2m)}{\left(z+\frac{t^{2}\mu_{c}b}{2m}\right)}^{2}\right]|{↑}_{z}\rangle \right.\\ & + & \left.\beta \exp\left[\frac{it\mu_{c}}{\hbar }\left(B_{0}+bz\right)\right]\exp\left[\frac{-1}{4(\sigma_{0}^{2}+it\hbar /2m)}{\left(z-\frac{t^{2}\mu_{c}b}{2m}\right)}^{2}\right]|{↓}_{z}\rangle \right\}. \end{array}$$

where ∇ is the vector differential operator, *m* is the mass of the particle, $\mu_{c}=g\frac{e\hbar }{4m_{e}}$ and *g* is the gyromagnetic ratio; see [5,6] for details. The magnetic field B of the SGE is an inhomogeneous magnetic field of the form $B=-bx\hat{i}+\left(B_{0}+bz\right)\hat{k}$, with $B_{0}$ a constant and *b* the strength of the inhomogeneity of the field.

## 4. Quantum Correlations and Nonlocality in the Stern–Gerlach Experiment

In this section, we calculate the quantum correlation and nonlocality arising in the SGE. To achieve that, we analytically work out the quantum correlation function C(z,θ) in the phase space, by redefining the correlation function proposed by Banaszek and Wódkiewicz [68,69,70] (see also [71]) in terms of the Wigner operator [72]. In other words, we define the correlation function in the following way:

Firstly, we define a correlation as:

(4) $$c\left(z,\theta \right)=\frac{1}{\pi \hbar }\langle \Psi |\hat{W}\left(z,p_{z}\right)\hat{\sigma }\left(\theta \right)|\Psi \rangle ,$$

where $p_{z}$ is the momentum in *z* and $\hat{W}\left(z,p_{z}\right)$ is the Wigner operator given by [72]; see also [73,74,75]:

(5) $$\hat{W}\left(z,p_{z}\right)=\frac{1}{2}\int_{-\infty }^{\infty }|z-\frac{q}{2}\rangle \exp\left(-\frac{iqp_{z}}{\hbar }\right)\langle z+\frac{q}{2}|dq,$$

$\hat{\sigma }\left(\theta \right)$ is the usual Pauli spin operator in an arbitrary direction θ, and *q* is a parameter. The 1/2 factor that multiplies the integral in Equation (5) derives from the parity operator defined by Royer [74], which is given by $\Pi_{rp}=\int e^{-i2ips/\hbar }r-s\rangle \langle r+s|ds$; by changing the variable *s* for *q*/2, you arrive at Equation (5). A possible path to deduce the Wigner operators is as follows: The definition of the Wigner function given by Wigner and collaborators is $P_{w}\left(q,p\right)=\frac{1}{\pi \hbar }\int e^{2ipy/\hbar }\langle q-y|\hat{\rho }|q+y\rangle dy$ [75]; from $P_{w}\left(q,p\right)$ and setting $\hat{\rho }=|\Psi \rangle \langle \Psi |$, we have $P_{w}\left(q,p\right)=\frac{1}{\pi \hbar }\int e^{2ipy/\hbar }\langle q-y|\Psi \rangle \langle \Psi |q+y\rangle dy=\frac{1}{\pi \hbar }\int e^{2ipy/\hbar }\langle \Psi |q+y\rangle \langle q-y|\Psi \rangle dy=\frac{1}{\pi \hbar }\langle \Psi |\left\{\int e^{2ipy/\hbar }|q+y\rangle \langle q-y|dy\right\}|\Psi \rangle$, and this is equal to the Royer definition and could explain the 1/2 factor in front of Equation (5); this renders ${\hat{W}}^{2}\left(z,p_{z}\right)=1$. In addition, to perceive the importance of the Wigner function for the understanding of quantum mechanics, see [75,76,77,78].

However, notice that the correlation c(z,θ) possesses dimensional factors that are originated because it is a correlation between the Wigner function and the Pauli operator. Hence, in order to avoid this dimensional factor and to present a correlation without dimension, we define the correlation C(z,θ) in the Stern–Gerlach experiment as:

(6) $$C\left(z,\theta \right)=\pi \hbar c\left(z,\theta \right)=\langle \Psi |\hat{W}\left(z,p_{z}\right)\hat{\sigma }\left(\theta \right)|\Psi \rangle .$$

Additionally, notice that the Wigner operator is, in fact, the parity operator around the points *z* and $p_{z}$ [72,74]. Here, we demonstrate that the SGE’s correlation function C(z,θ) violates the Bell’s inequality [68]. In the case of entangled pure states, this violation of Bell’s inequality is usually interpreted as the signature of nonlocality in quantum mechanics [68,69,79,80]; then, based on the preceding, in this paper, we restrict ourselves to this interpretation only. However, see [81] for a different interpretation.

In addition, notice that, as was pointed out by Ferraro and Paris, the amount of violation of Bell’s inequality specifically depends on the kind of Bell operators used to test it [82]. Furthermore, the degree of quantum nonlocality depends on the type of entangled state [80]. For example, with regard to the approach of Banazek and Wódkiewicz, the maximal violation attained for a two-mode squeezed state is approximately 2.32 [68,69]; though, for the formalism proposed by Chen et al. [80], the maximal violation attained, for the same two-mode squeezed states, reaches the maximum value, i.e., $\approx 2\sqrt{2}$ [79]. See also [82] for an instructive discussion of nonlocality in continuous variables for two and three modes.

### 4.1. A Pure State

In this section, we consider a pure state for the position and the spin that traverses an SGE. The effect of the SGE is to produce an entangled state between the internal and the external degrees of freedom, as given in [5,6]. Then, the state coming out from the SGE, given in the previous section, can be written as follows:

(7) $$|\psi (t)\rangle =c_{0}\left(x,y,t\right)\frac{1}{\sqrt{2}}\left(|\varphi_{+}\left(t\right)\rangle |{↑}_{z}\rangle +|\varphi_{-}\left(t\right)\rangle |{↓}_{z}\rangle \right),$$

where $c_{0}\left(x,y,t\right)$ are the variables on *x* and *y* dimensions that appear in the previous section or in [5,6], which are in concordance with the definition $A_{1}=\exp\left(-\frac{it^{3}\mu_{c}^{2}b^{2}}{m\hbar }\right){\left(\frac{\sigma_{0}}{\sqrt{2\pi }}\right)}^{\frac{1}{2}}{\left(\sigma_{0}^{2}+\frac{i\hbar t}{2m}\right)}^{-\frac{1}{2}}$, that is:

(8) $$\begin{array}{cc} c_{0}\left(x,y,t\right) & =\exp\left(\frac{5it^{3}\mu_{c}^{2}b^{2}}{6m\hbar }\right)\left[\frac{\sigma_{0}}{{\left(2\pi \right)}^{1/2}}\right]{\left(\sigma_{0}^{2}+\frac{i\hbar t}{2m}\right)}^{-1}\exp\left(-\sigma_{0}^{2}k_{y}^{2}\right)\\ & \times \exp\left[\frac{i4y\sigma_{0}^{2}k_{y}}{4(\sigma_{0}^{2}+it\hbar /2m)}\right]\exp\left[\frac{-(x^{2}+y^{2}-4\sigma_{0}^{4}k_{y}^{2})}{4(\sigma_{0}^{2}+it\hbar /2m)}\right], \end{array}$$

we have set the constants α and β of Equation (1) equal to $1/\sqrt{2}$, where the position states $|\varphi_{+}\left(t\right)\rangle$ and $|\varphi_{-}\left(t\right)\rangle$ are such that:

(9) $$\begin{array}{c} \langle z|\varphi_{+}\left(t\right)\rangle =A_{1}\exp\left[-\frac{it\mu_{c}}{\hbar }\left(B_{0}+bz\right)\right]\exp\left[-\frac{{\left(z+\frac{t^{2}\mu_{c}b}{2m}\right)}^{2}}{4\left(\sigma_{0}^{2}+\frac{it\hbar }{2m}\right)}\right], \end{array}$$

(10) $$\begin{array}{c} \langle z|\varphi_{-}\left(t\right)\rangle =A_{1}\exp\left[\frac{it\mu_{c}}{\hbar }\left(B_{0}+bz\right)\right]\exp\left[-\frac{{\left(z-\frac{t^{2}\mu_{c}b}{2m}\right)}^{2}}{4\left(\sigma_{0}^{2}+\frac{it\hbar }{2m}\right)}\right]. \end{array}$$

Notice that $\langle z|\varphi_{+}\left(t\right)\rangle$ and $\langle z|\varphi_{-}\left(t\right)\rangle$ are not orthogonal; however, they are properly normalized in the variable *z* when taking into account the constant $A_{1}$. Henceforward, in order to facilitate this analysis, we focus on the single position dimension *z*; in other words, we do not take into account the other two dimensions. As a consequence, in the subsequent paragraphs, we will just employ the coordinate *z*.

Then, using Equation (7), we calculate C(z,,θ) as:

(11) $$\begin{array}{ccc} C(z,\theta ) & = & \langle \varphi_{+}\left(t\right)|\hat{W}\left(z,p_{z}\right)|\varphi_{+}\left(t\right)\rangle \langle {↑}_{z}|\hat{\sigma }\left(\theta \right)|{↑}_{z}\rangle +\langle \varphi_{+}\left(t\right)|\hat{W}\left(z,p_{z}\right)|\varphi_{-}\left(t\right)\rangle \langle {↑}_{z}|\hat{\sigma }\left(\theta \right)|{↓}_{z}\rangle \\ & + & \langle \varphi_{-}\left(t\right)|\hat{W}\left(z,p_{z}\right)|\varphi_{+}\left(t\right)\rangle \langle {↓}_{z}|\hat{\sigma }\left(\theta \right)|{↑}_{z}\rangle +\langle \varphi_{-}\left(t\right)|\hat{W}\left(z,p_{z}\right)|\varphi_{-}\left(t\right)\rangle \langle {↓}_{z}|\hat{\sigma }\left(\theta \right)|{↓}_{z}\rangle . \end{array}$$

Equation (11) establishes the quantum correlations emerging from the SGE, and after lengthy calculations, we arrive at the next expression:

(12) $$\begin{array}{ccc} C(z,\theta ) & = & \frac{\cos\theta }{2}\left(\exp\left\{-\frac{\sigma_{0}^{2}{\left(z+\frac{t^{2}\mu_{c}b}{2m}\right)}^{2}}{2\left(\sigma_{0}^{4}+\frac{\hbar^{2}t^{2}}{4m^{2}}\right)}-\frac{2\left(\sigma_{0}^{4}+\frac{\hbar^{2}t^{2}}{4m^{2}}\right)}{\sigma_{0}^{2}}{\left[\frac{p_{z}}{\hbar }+\frac{t\mu_{c}b}{\hbar }-\frac{\hbar t\left(z+\frac{t^{2}\mu_{c}b}{2m}\right)}{4m\left(\sigma_{0}^{4}+\frac{\hbar^{2}t^{2}}{4m^{2}}\right)}\right]}^{2}\right\}\right.\\ & - & \left.\exp\left\{-\frac{\sigma_{0}^{2}{\left(z-\frac{t^{2}\mu_{c}b}{2m}\right)}^{2}}{2\left(\sigma_{0}^{4}+\frac{\hbar^{2}t^{2}}{4m^{2}}\right)}-\frac{2\left(\sigma_{0}^{4}+\frac{\hbar^{2}t^{2}}{4m^{2}}\right)}{\sigma_{0}^{2}}{\left[\frac{p_{z}}{\hbar }-\frac{t\mu_{c}b}{\hbar }-\frac{\hbar t\left(z-\frac{t^{2}\mu_{c}b}{2m}\right)}{4m\left(\sigma_{0}^{4}+\frac{\hbar^{2}t^{2}}{4m^{2}}\right)}\right]}^{2}\right\}\right)\\ & + & \sin\theta \exp\left[-\frac{\sigma_{0}^{2}z^{2}}{2\left(\sigma_{0}^{4}+\frac{\hbar^{2}t^{2}}{4m^{2}}\right)}-\frac{2\left(\sigma_{0}^{4}+\frac{\hbar^{2}t^{2}}{4m^{2}}\right)}{\sigma_{0}^{2}}{\left(\frac{p_{z}}{\hbar }-\frac{\hbar tz}{4m\left(\sigma_{0}^{4}+\frac{\hbar^{2}t^{2}}{4m^{2}}\right)}\right)}^{2}\right]\\ & & \cos\left[-\frac{2t\mu_{c}}{\hbar }\left(B_{0}+bz\right)+\frac{\hbar t^{3}\mu_{c}bz}{4m^{2}\left(\sigma_{0}^{4}+\frac{\hbar^{2}t^{2}}{4m^{2}}\right)}+\frac{t^{2}\mu_{c}b}{m}\left(\frac{p_{z}}{\hbar }-\frac{\hbar tz}{4m\left(\sigma_{0}^{4}+\frac{\hbar^{2}t^{2}}{4m^{2}}\right)}\right)\right] \end{array}$$

In this case, Equation (12) represents the correlation function that arises between the direction of the spin and the *z* position of the atom; it exhibits the interference and the entanglement of the internal and external degrees of freedom, and it shows that there are values where the correlation is minimum and some values where this correlation is maximum. In essence, this result shows that the measurement outcomes of the *z* position may depend nonlocally on the measurement outcome of the internal degree and vice versa. In other words, the dichotomic observables in this case are the parity operator given in Equation (5) and the spin of the atom in an arbitrary direction. Moreover, Equation (12) has two terms: the first term has two Wigner functions (if multiplied by πℏ), which are displaced by a term $d=t^{2}\mu_{c}b/2m$, and they move in opposite direction in position space with velocity proportional to $\mu_{c}b/\hbar$, whereas the second one has an oscillating cosine term, which is responsible for the oscillations. Additionally, it is important to mention that Equation (12) depends on six parameters: the time *t*, the position *z*, the strength of the divergence of the magnetic field *b*, the momentum $p_{z}$, the initial width of the wave packet $\sigma_{0}$ and the angle θ.

**Figure 1:**
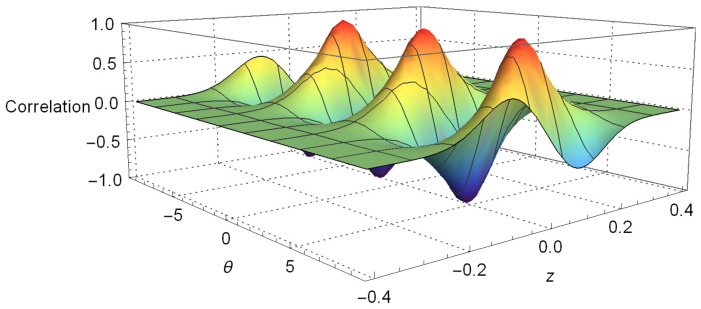
A plot of the correlation function between *z* and θ given by Equation (12). To obtain this plot, we have employed the following values: first, we set πℏ=1 and m=1; then, we set $\sigma_{0}=0.005$, $\mu_{c}b/2=2.2$, $p_{z}=0.01$ and time t=0.2.

We have plotted Equation (12) on Figure 1, where we take $p_{z}$ as a constant; we set πℏ=1 and m=1 in a very similar way as is carried out in [11,70]. This figure allows us to see the oscillatory behavior of the correlation function with variables *z* and θ predicted in Equation (12). Furthermore, there, we can notice how the oscillations in *z* decay very fast when its values are increased, until this effect is hardly appreciated. In the same way, the negativity of the Wigner function is also perceived, which is commonly associated with some kind of nonclassical behavior, although some care must be taken when using this interpretation of the negativity because it involves the spin variable also. This issue is associated with the definition of the Wigner function for finite dimensional Hilbert space [83,84,85,86].

To conclude this section, it is important to remark that the correlation function in Equation (12) can be put in a very similar way to the one of the correlation function for the Schrödinger cat state seen in Wódkiewicz’s article [68], so that the same conclusions at which he arrives regarding the displacement *D* still remain valid.

### 4.2. Violation of Bell’s Inequalities

In hidden variables theories, quantum correlations are thought to arise from the average of the correlation function with respect to the hidden variables λ over statistical distributions. In particular, for the case of SGE, we apply the analysis implemented by Wódkiewicz where the average is given by [68]; see also [79,80]:

(13) $$C\left(z,\theta \right)=\int d\lambda_{ext}\int d\lambda_{int}W\left(z,\lambda_{ext}\right)\sigma \left(\theta ,\lambda_{int}\right)P\left(\lambda_{ext},\lambda_{int}\right),$$

where $\lambda_{ext}$ and $\lambda_{int}$ are hidden variables of the external and internal degrees of freedom, respectively, $W(z,\lambda_{ext})=\pm 1$ represents the parity operator complemented with the hidden variable $\lambda_{ext}$ and its values ±1 are the local realities of the external degree of freedom. On the other hand, $\sigma (\theta ,\lambda_{int})=\pm 1$ represents the Pauli operator complemented with the hidden variable $\lambda_{int}$, and its values ±1 represent the local realities of the internal degree of freedom. Finally, $P(\lambda_{ext},\lambda_{int})$ is the density distribution of the hidden variables. According to the Clauser–Horne–Shimony–Holt (CHSH) analysis of Bell’s inequalities [87], this correlation should obey the following inequalities:

(14) $$-2\leq C\left(z^{\prime },\theta^{\prime }\right)+C\left(z^{\prime },\theta \right)+C\left(z,\theta^{\prime }\right)-C\left(z,\theta \right)\leq 2.$$

Thus, a violation of these inequalities by quantum mechanical correlations arises from the nonlocality of quantum phenomena. It is important to emphasize that *C* represents the correlations that are the product of the hidden variables’ average in a hidden variables theory.

On the other hand, from Equation (12), we can calculate the function $B_{z}$ for the quantum correlations of the SGE as follows:

(15) $$\begin{array}{c} B_{z}=C\left(z^{\prime },\theta^{\prime }\right)+C\left(z^{\prime },\theta \right)+C\left(z,\theta^{\prime }\right)-C\left(z,\theta \right). \end{array}$$

Note that, given the form of Equation (14), we may consider the correlations between *z* and θ for Equation (15) by taking $p_{z}$ as a constant in Equation (12) to obtain Equation (15).

**Figure 2:**
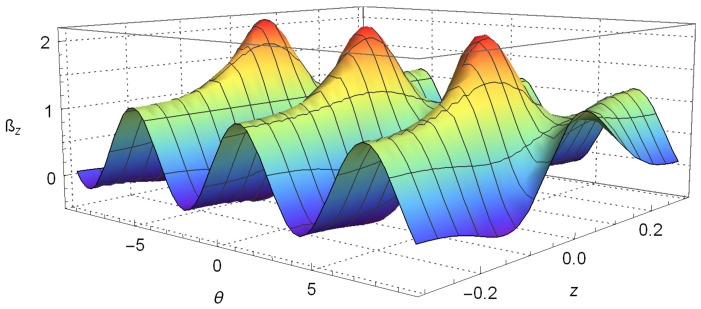
A plot of the function ${}_{z}$ given by Equation (15) considering $z^{\prime }=0.08$ and $\theta^{\prime }=\pi /2$. Once more, we set πℏ=1, m=1, $\sigma_{0}=0.005$, $\mu_{c}b/2=2.2$, $p_{z}=0.01$ and time t=0.2.

**Figure 3:**
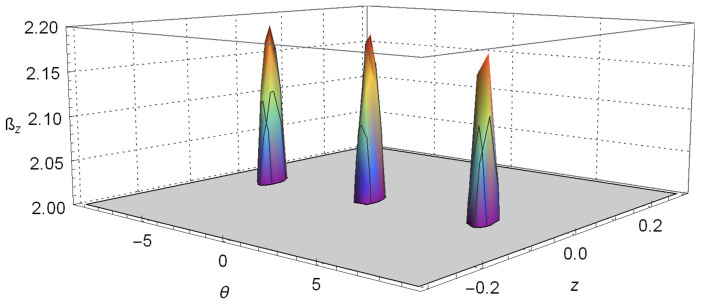
A close up of the region of Figure 2 where the violation of Bell’s inequality is perceived.

Plots of Equation (15), setting $z^{\prime }=0.08$ and $\theta^{\prime }=\pi /2$, are given in Figure 2 and Figure 3. As in the last section, we set the constants by making $\mu_{c}b/2=2.2$, t=0.2, $\sigma_{0}=0.005$ and considering πℏ=1 and m=1. These plots clearly show the violation of Bell’s inequalities given by Equation (15). This means that the quantum correlation arising from Equation (12) and shown in Figure 1 cannot be explained by local influences or local causes.

It is important to mention that the violation of the CHSH inequality, shown in Figure 3, does not reach the maximum value $2\sqrt{2}$. This is due to two factors: first, we found it by conjecturing values for the parameters that place us near the violation; then, we varied the values to find the violation. Notice that there could probably exist values where the violation might be higher. Second, as the states of the external degree of freedom are Gaussian, it seems that we can apply the explanation given by Haug et al. [70] stating that the Gaussian form of the correlation function smooths the CHSH-correlation, and therefore, it reduces the maximal possible value of the correlation.

## 5. Conclusions

Quantum mechanics is a fascinating field; nonetheless, the core ideas, like quantum nonlocality and “disturbance”, are difficult concepts to grasp. In fact, the concept of disturbance, caused by the measurement process, which is responsible for one of the interpretations of the Heisenberg uncertainty principle, is not fully understood yet, and it is under investigation, as well [88]. In this case, disturbance refers to the change and perturbation produced by the measurement process; see [88] and the references therein. Quantum nonlocality is captivating, as well, and the fact of analyzing nonlocality by using the highly significant SGE could lead to understanding this concept better.

With this in mind, in this article, we have studied the correlations arising from the evolution of a pure state in the SGE, using the results given in [5,6] for the quantum mechanical evolution in the SGE and the approach given in [68] for testing nonlocality. In this way, we revealed that the SGE presents a nonlocal behavior, something that has never been thoroughly studied in the literature before. Once the correlations and nonlocality in the SGE have been characterized, we would like to conclude this paper by proposing the SGE as a scaffolding to introduce these concepts to students of quantum mechanics, as its experimental features can make the exposition of these concepts especially intuitive; additionally, this approach could open new investigations in consecutive SGEs or in quantum roulettes.
